# Editorial: Smart materials and additive manufacturing for functional tissue engineering

**DOI:** 10.3389/fbioe.2026.2019823

**Published:** 2026-10-29

**Authors:** Yifan Wang, Zheng Wang

**Affiliations:** Department of Orthopedics, Chinese PLA General Hospital, Beijing, China

**Keywords:** additive manufacturing, bioinks, functional validation, smart biomaterials, tissue engineering

Functional tissue engineering asks more of a construct than anatomical resemblance: it must serve a defined biological purpose and retain that capacity after fabrication. Responsive materials can couple local cues to therapeutic release or cell regulation, while additive manufacturing can organize cells and matrices in controlled architectures. This Research Topic examines how material design, manufacturing, and validation can be connected so that a construct’s intended function is measurable and reproducible. Its eight contributions span design frameworks, experimental scaffolds, evidence mapping, and clinical use cases.

At the formulation stage, Zhang et al. review artificial intelligence (AI) across hydrogel bioink design, printability prediction, process monitoring, and construct validation. They distinguish established applications from prospective closed-loop bioprinting and emphasize external validation and biological endpoints. Ding et al. extend the design problem to functional peptides: foundation models can prioritize antimicrobial, targeting, and cell-instructive candidates, but activity must survive immobilization, crosslinking, and printing. Together, these reviews frame AI as a tool for choosing testable materials, rather than evidence of construct performance.

Evidence needs to remain traceable after candidates enter biological studies. Li et al. assemble SenescenceBioKG from 283 full-text studies to link material features, cellular targets, mechanisms, and regenerative outcomes within the same reports. Their analysis identifies recurring responsive designs but also sparse organoid, large-animal, and clinical evidence. This approach makes gaps in validation visible without treating co-occurrence as proof of causation.

The challenge of combining therapeutic and regenerative functions is especially clear in bone cancer. Xu et al. review bifunctional scaffolds designed to suppress osteosarcoma while supporting bone regeneration, including stimuli-responsive release and patient-specific printing. In a complementary opinion, Li et al. propose AI-guided photoresponsive nanoplatforms that could adjust treatment intensity as tumor and repair needs change. The latter is a conceptual direction; its proposed feedback control still requires experimental demonstration. Both articles expose the need to test cancer control and bone repair together, over relevant timescales.

A direct experimental example comes from Tian et al., who functionalize an electrospun scaffold with polydopamine-bound curcumin for infected wounds. Their *in vitro* assays and infected rat wound model report antibacterial, antioxidant, immunomodulatory, and pro-angiogenic effects alongside improved healing. This study illustrates how several intended functions can be evaluated in one disease-relevant construct, although broader reproducibility and clinical performance remain open questions.

Disease context also determines which evidence matters. Wang et al. map 596 glaucoma-related biomaterial publications and find an uneven shift from drug delivery toward smart materials, additive manufacturing, and functional tissue models. Their findings point to a need for disease-specific endpoints rather than assuming that a sophisticated material confers functional benefit. In general surgery, Lou et al. review responsive hydrogels, bioinks, and patient-specific scaffolds against practical failure modes such as leakage, infection, and impaired tissue integration. Their proposed GS0–GS5 hierarchy connects material tests to the conditions of surgical use.

Across these contributions, the common requirement is an explicit chain from a material or computational choice to a manufacturable construct and then to a relevant biological outcome. A responsive signal needs a defined threshold and duration; a printable formulation needs documented process conditions and quality controls; a predicted benefit needs prospective testing in the intended tissue setting. The Research Topic therefore places functional validation and reproducibility alongside material novelty. Connecting these elements should make the next-generation of smart, additively manufactured tissue constructs easier to compare and more credible as candidates for translation.

